# The impact of interdisciplinary cardiac arrest teams on paramedic practise: an interpretive qualitative study using reflexive thematic analysis

**DOI:** 10.1016/j.resplu.2026.101317

**Published:** 2026-04-07

**Authors:** Jacob Tant, Jackie Buckthought, Ashleigh Scianski, Izabelle Xu, Brian Burns, Natalie Kruit

**Affiliations:** a Aeromedical Operations, New South Wales Ambulance, Sydney, New South Wales, Australia; b School of Health Sciences, Western Sydney University, Sydney, New South Wales, Australia; c Clinical Operations, New South Wales Ambulance, Sydney, New South Wales, Australia; d Faculty of Medicine and Health, University of Sydney, Sydney, New South Wales, Australia

**Keywords:** OHCA, ECPR, Paramedic, Interprofessional teams, Multidisciplinary teams

## Abstract

**Objectives:**

This study examined paramedics’ perceptions of the influence of a specialist out-of-hospital cardiac arrest (OHCA) extracorporeal cardiopulmonary resuscitation (ECPR) team on their resuscitation practise and professional development.

**Methods:**

An interpretive qualitative methodology was employed. A purposive sample of 379 NSW Ambulance paramedics who attended an OHCA alongside the PRECARE team was invited to complete an anonymous online survey, of whom 67 participated. The survey included demographic questions for sample context and open-ended, non-leading questions designed to capture in-depth insights. Reflexive thematic analysis was conducted collaboratively by multiple authors, guided by the principle of theoretical sufficiency.

**Results:**

Five themes were identified: Mentorship, Empowerment, Understanding and Development, Cause-Directed Care and Collaborative Care. Participants frequently described PRECARE’s real-time mentoring and post-event debriefs as influential in shaping their clinical confidence and skill development. Working alongside PRECARE teams was reported to enhance participants’ understanding of OHCA physiology and pathophysiology, evidence-based interventions, and the importance of cause-directed and individualised care. Paramedics also described changes in motivation, accountability, and non-technical skills such as leadership and communication. Alongside these perspectives, some participants described challenges related to role clarity, leadership dynamics, and operational priorities.

**Conclusions:**

The integration of specialist interdisciplinary OHCA teams was perceived to influence paramedics’ clinical practise and professional development by contributing to a psychologically safe working environment and providing experiential learning opportunities, including real-time mentorship and reflective post-event debriefing. This interdisciplinary collaboration was associated with increased confidence, perceived strengthening of non-technical skills, and a shift toward more nuanced, cause-directed care, while also highlighting the importance of clear role delineation and alignment of operational priorities when integrating specialist teams into established EMS systems.

## Introduction

The deployment of traditionally hospital-based resuscitative interventions into the pre-hospital setting via a specialist Interdisciplinary cardiac arrest response team may improve access to advanced life-saving therapies and potentially improve outcomes for patients experiencing out-of-hospital cardiac arrest (OHCA).^1^ Beyond direct clinical care, such specialist teams offer unique opportunities for interdisciplinary collaboration and real-time mentorship of paramedics by senior clinicians with concentrated exposure to OHCA cases. The influence of this model may extend beyond individual OHCA cases, potentially contributing to a culture of continuous professional development and system-level quality improvement.

Existing literature examining interdisciplinary OHCA response teams has largely focused on the feasibility and clinical outcomes associated with advanced prehospital interventions such as extracorporeal cardiopulmonary resuscitation (ECPR).^1^, ^2^, ^3^, ^4^ Less attention has been given to how working alongside specialist OHCA teams influences paramedics’ clinical practise and learning. Exploring these perspectives may provide insight into how specialist teams contribute to interdisciplinary learning and broader improvements in resuscitation practise within emergency medical services (EMS).

This study examines paramedics’ perceptions of the impact of a specialist OHCA ECPR interdisciplinary team on their practise in delivering high-quality resuscitation. To our knowledge, this is the first study to explore how exposure to a prehospital ECPR team influences paramedic cardiac arrest practise.

## Methods

### Context

New South Wales Ambulance (NSWA) is the jurisdictional EMS provider for the state of New South Wales (NSW), Australia, serving a population of approximately 8.2 million people. As one of the largest EMS providers in the southern hemisphere, NSW Ambulance responds to over 1.2 million incidents annually.^5^ The organisation operates a tiered clinical model that includes primary care paramedics, paramedic specialists such as intensive care paramedics (ICP), and aeromedical teams composed of prehospital and retrieval medicine (PHRM) physicians, critical care paramedics (CCPs) and flight nurses. This structure enables adaptive clinical responses tailored to scene complexity and patient acuity.

All paramedics are trained in both basic and advanced life support (BLS and ALS) and high-performance cardiopulmonary resuscitation (HPCPR) as a system-wide standard of care. ICPs receive additional credentialling across a broader range of critical care interventions, which includes endotracheal intubation and intraosseous access. Approximately 3500 OHCA cases receive attempted resuscitation by NSWA paramedics each year^6^ and are designated the highest clinical priority, typically prompting a multi-team response including at least one ICP.

In August 2023, NSWA commenced the PRECARE study (Prehospital ECPR for refractory cardiac arrest), designed to assess the feasibility, safety, and impact of delivering pre-hospital ECPR to patients with refractory OHCA.^3^ Outside the PRECARE study, NSWA aeromedical teams do not routinely deploy to typical adult OHCA cases. As part of this trial, a dedicated advanced cardiac arrest response team, consisting of two PHRM physicians and one CCP is deployed three days per week within the Sydney metropolitan area. Prior to and during the study period, paramedics were offered voluntary educational opportunities to familiarise them with the PRECARE model of care, including online question-and-answer sessions and practical demonstrations of the PRECARE prehospital ECMO workflow. Given ECPR is a time-sensitive procedure, PRECARE teams are tasked rapidly and liberally to ensure early arrival to potential ECPR-eligible cases. Naturally, this has resulted in PRECARE team involvement in many non-ECPR-eligible cases. In these instances, the PRECARE teams work alongside paramedic teams and provide complementary clinical support which includes a suite of advanced resuscitative diagnostics and therapeutics, such as transesophageal echocardiography (TOE), invasive blood pressure (IBP) monitoring, mechanical ventilation and post-ROSC neurocritical care. On average, the team attends one to two OHCA cases per shift.^3^

### Study design

We used an interpretive qualitative methodology underpinned by a relativist ontology and a blended epistemological stance drawing on both subjectivism and constructivism. Data were collected using an open-ended survey, and analysis was conducted using reflexive thematic analysis (RTA) as described by Braun and Clarke.^7^, ^8^, ^9^ The study was reported in line with the Standards for Reporting Qualitative Research (SRQR).^10^

### Sampling Strategy

Purposive sampling was employed to recruit any NSWA paramedic who had personally attended an OHCA alongside PRECARE clinicians. The paramedics’ names were identified from electronic medical records and their staff contact emails were extracted from the NSWA Outlook (Microsoft, USA) address book. Paramedics were emailed an online survey with instructions that emphasised their involvement in the study was optional and that their responses would be anonymous. The survey remained active for a one-month period, from 27 February 2025 to 27 March 2025, and the paramedics received weekly reminders to participate if they wished. Completion of the online survey implied consent, and only survey responses that included a minimum of one answered open-ended question were included in the study.

### Data collection

Data were collected using an online survey built on the Qualtrics (https://www.qualtrics.com) platform. The survey design was modelled on recommendations made by Braun and Clarke,^7^, ^8^, ^9^ Simpson,^11^ and Thomas et al.^12^ Online surveys lend themselves to interpretivist projects as they can be used to collect large amounts of information from a wide range of participants, including opinions, experiences, and practises.^12^ For participants, online surveys offered flexible engagement^7^, ^13^ and greater control regarding the extent of their participation and autonomy.^12^, ^14^

The survey was created to be accessible in terms of length, clarity, and guidance. The survey included four basic demographic questions that allowed for the contextualization of the sample,^12^ followed by five open-ended questions. The open-ended questions themselves were designed to address the research aims and to be non-leading in nature, with the final open-ended question acting as a ‘clean-up’ question.^7^, ^12^ The survey questions can be found in Table 1.

**Table 1:** Online survey questions.

**Question number**	**Survey question**
Q1	What is your paramedic role?
Q2	How many years have you worked as a paramedic?
Q3	Please estimate the average number of cardiac arrests that you attend each year.
Q4	What is the highest level of education that you have completed?
Q5	Please describe how the PRECARE medical team has impacted your understanding of cardiac arrests.
Q6	Has your experience working with PRECARE medical teams influenced your ongoing approach to managing cardiac arrests, with consideration of the technical and non-technical skills involved? Please provide examples from any cardiac arrests you’ve attended to since working with PRECARE.
Q7	How would you describe the impact of the real-time support you received from PRECARE medical teams whilst attending a cardiac arrest? Please provide as much detail as possible.
Q8	Has working alongside PRECARE medical teams led you to identify personal or team-wide educational needs relating to cardiac arrest management? How did PRECARE help you recognise these needs?
Q9	(OPTIONAL) Has working with PRECARE medical teams impacted you or your paramedic practise in any ways that have not been covered? Is there anything else you would like to share about your experience working with PRECARE medical teams?

A pilot of the study was conducted prior to implementation with a sample of paramedics who had not worked alongside PRECARE teams. This pilot ensured the online survey itself worked as intended and that the survey questions were clear and non-leading in their design.

### Data analysis

A reflexive thematic analysis (RTA) was conducted using the analytical software NVivo (QSR International Pty Ltd., 2023), guided by Braun and Clarke^5^, ^6^, ^7^ and Byrne.^12^ RTA underlines the researcher’s active role in analysis, highlighting how the interpretations made by the researcher is influenced by their theoretical assumptions and skills as much as the dataset itself. For this project, multiple authors collaborated on the RTA thereby improving the rigour of the process and achieving a richer interpretation of meaning through a multi-pronged approach and shared sense-making.

The collaborative RTA was conducted by authors AS (female, paramedic, academic) and JT (male, critical care paramedic, academic). AS and JT were chosen to complete the RTA as both authors work for NSWA within the PRECARE teams' response area, so have adequate contextual understanding of the research aims, but have not personally worked within or alongside PRECARE teams.

The RTA followed a six-step process: (1) data familiarisation, (2) initial code generation, (3) the generation of themes, (4) the review of generated themes, (5) defining and naming themes, and (6) reporting. The analysts moved forwards and backwards through these steps as required. Coding was predominantly inductive, so that identified codes were reflective of the dataset as communicated by the participants,^5^, ^12^ though a degree of deduction was required to ensure results related to the research aims.^12^ The analysts employed both latent and semantic coding, generating codes based on the author’s interpretations of the underlying assumptions and ideas of the participants, as well as the surface meaning of their words. The analysts compared and critically analysed their coding at length and worked together to generate themes. Following this, member checking was employed to further the trustworthiness of the RTA. All participants were emailed a summary of the results and were given a week-long period to reply. Two participants responded requesting to view the dataset.

For this study, theoretical sufficiency was judged in relation to the narrow scope of the research question, the richness of the dataset, the depth of the developed themes, and linkages made to existing theories, concepts and models.^14^, ^15^ If theoretical sufficiency had not been achieved using the online survey a second collection method would have been employed.

### Ethics

This study complies with the National Statement on Ethical Conduct and Research.^16^ Ethical approval for this study was granted by the Sydney Local Health District HREC (HREC reference numbers: X23-0150 and 2023/ETH00767).

## Results

### Background context

Of 379 paramedics who had clinical contact with PRECARE and were emailed the survey, a total of 67 (17.8%) participated in the study. The characteristics of these participants are outlined in Table 2. Individual participant demographic details can be found in Appendix.

**Table 2:** Participant characteristics.

**Characteristic**	***N* (%)**
**Paramedic level/specialty**	
Trainee	1 (1.49)
Paramedic	22 (28.57)
Intensive care	45 (58.44)
Extended care	8 (10.39)
Special ops & rescue	1 (1.49)
**Highest level of education**	
High school	1 (1.49)
Associate’s degree	8 (11.94)
Bachelor’s degree	36 (53.73)
Postgraduate certificate or degree	17 (25.37)
Master’s degree	5 (7.46)

	**Median (IQR)**

Estimated OHCA cases attended per year	12 (8–25)
Years of Paramedic Experience	10 (5–17)

Note: *N* = count of participants. For continuous variables, median and interquartile range (IQR) are reported. Some participants held more than one specialty, so totals exceeded 100%.

The median response rate for all open-ended questions (Q5–9) was 4.6 out of 5. Individual response rates were 98.5% for Q5, 95.5% for Q6, 92.5% for Q7, 92.5% for Q8, and 38.8% for Q9. Responses were generally substantive and narrative in form. Most participants provided sentence- or paragraph-length answers, frequently incorporating multiple reflective points within a single answer. Only a small number of participants provided single-word or brief phrase responses.

### Introduction to themes

**Fig. 1:**
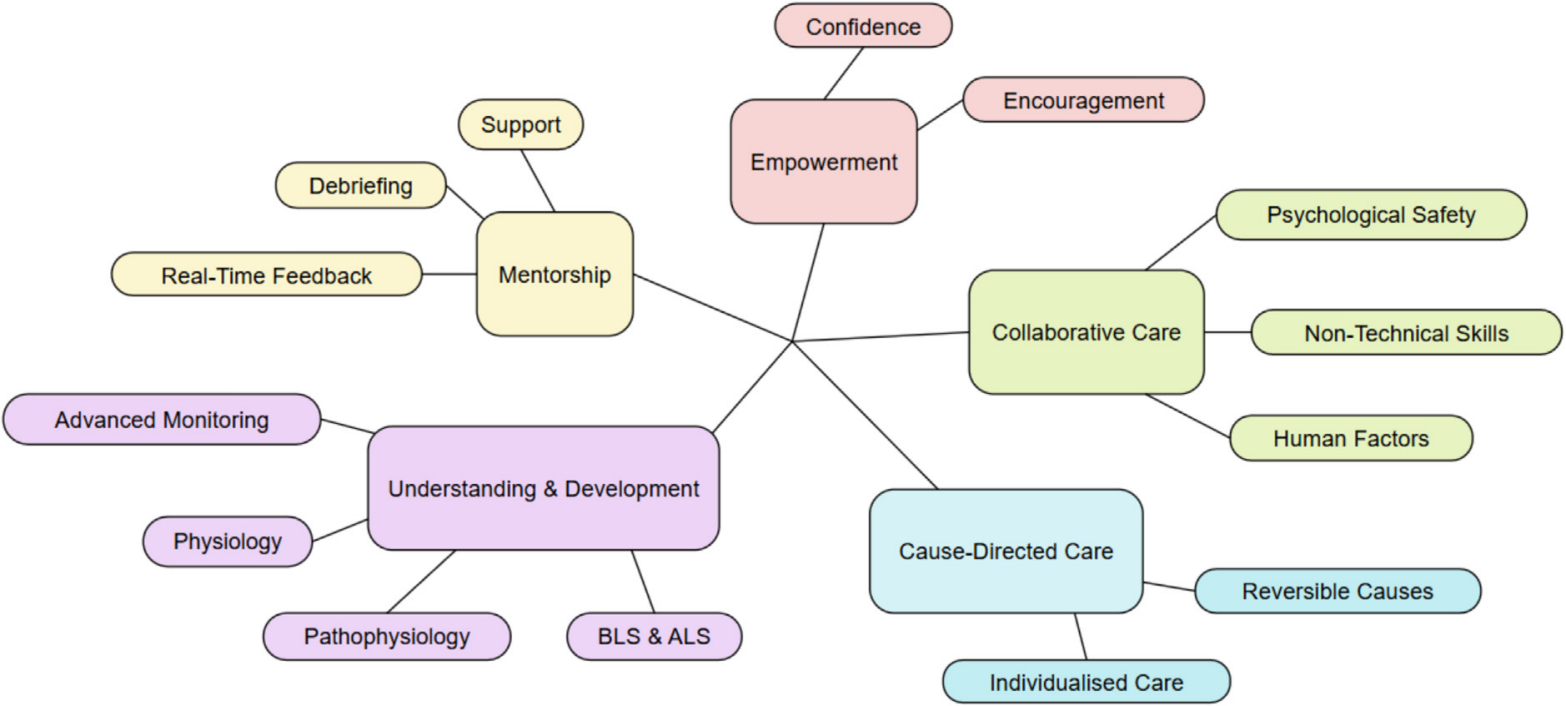
**Thematic map illustrating the relationship between the major themes and subthemes identified in the analysis**.

Five themes and associated sub-themes were identified: Mentorship, Empowerment, Understanding and Development, Cause-Directed Care and Collaborative Care. These themes are mapped out in Fig. 1.

### Theme one: mentorship

This theme describes how participants experienced mentorship from senior clinicians during OHCA cases, including real-time guidance, feedback, and experiential learning.

Participants appreciated how PRECARE teams integrated with paramedic crews during prehospital cases. PRECARE members were noted to be friendly and respectful, contributing to a collaborative working environment that promoted psychological safety and high-quality care.

*“…The [team] created an inclusive and supportive environment. Far from arriving on scene and taking over [management of the patient], they integrate themselves as appropriate, closely consider the crew’s handovers, and become “part of the team*…*””*

*“The PRECARE medical team supported my decision making and trusted my judgement and clinical capability. This provided me both clinical and psychological support.”*

Participants felt reassured when working alongside PRECARE clinicians. Paramedic leaders valued the dynamic leadership model fostered by PRECARE, highlighting its ability to balance shared cognitive load, active followership, crew resource management, and assistance with clinical reasoning and decision-making. Participants felt supported with the practical application of their skills and equipment. They highlighted improvement in their technical safety, operational effectiveness and adherence to evidence-based practises.

*“The PRECARE team has been incredibly supportive both on and off scene, providing guidance during critical moments and valuable feedback afterwards.”*

Participants felt valued when working with PRECARE. They expressed satisfaction that they were providing best possible care as a team, and that their individual roles and responsibilities were contributing positively towards their patients' outcomes and ongoing healthcare journeys.

*“The real-time support I received from the PRECARE team during a cardiac arrest was invaluable and has had a lasting impact on my practise.”*

Working alongside a senior interdisciplinary team provided opportunities for experiential learning that is traditionally limited or unavailable to paramedics. Participants engaged in real-time learning by observing the PRECARE teams processes, raising uncertainties or concerns as they arose, and receiving immediate feedback on their clinical decisions and interventions. Participants reported that this learning enhanced their reactivity and adaptability in complex and rapidly changing clinical situations. Participants reported this as being particularly impactful for junior paramedics and paramedic leaders.

*“Real-time mentoring in the pre-hospital environment is incredibly difficult due to the high-pressure nature of these situations, and it is something that traditional simulations can never fully replicate. Having someone… experienced, knowledgeable, and open to sharing… made a tremendous difference.”*

*“Their real-time mentoring enhanced my understanding of the clinical situation and improved my decision-making in the moment.”*

*“It was a complex case, but [the PRECARE clinician] was exceptional in her capacity to explain to the ICP (in real time) … It was incredibly rewarding and the learning generated triggered a greater understanding.”*

Following each OHCA case the PRECARE teams routinely conduct hot debrief sessions with the attending paramedic crews. Participants described these debriefs as reinforcing collaboration and psychological safety and providing additional opportunities for PRECARE teams to explain and rationalise complex critical care concepts. Frequent topics raised within the data included pathophysiology, atypical physiological presentations, pharmacological and technical interventions. Participants highlighted how these debriefs deepened their understanding, inspired them to reflect on their practises, and motivated them to pursue self-directed learning.

*“I truly believe debriefing jobs is critical to welfare and to learning, and I have worked hard over the years to improve this aspect of my practise. But watching the [PRECARE] team have an approach to debrief that was both educational and empowering had a big impact on me.”*

*“They gave feedback that instilled confidence within the team, yet acknowledged what challenges we could come across as a team in assisting the PRECARE team, and gave us insight into addressing these. This benefited my learning as a clinical leader greatly.”*

Collectively, these accounts illustrate how real-time mentorship within active resuscitation environments created opportunities for experiential learning that participants perceived as otherwise difficult to replicate.

### Theme two: empowerment

This theme captures how these experiences influenced participants’ confidence, professional motivation, and engagement with clinical practise.

At the scene, it was perceived that the presence of PRECARE teams supported decision making through enhanced situational awareness and shared clinical expertise. The poise and assertiveness of paramedic leaders was bolstered leading to the enhanced development of team goals and more efficient delegation of tasks. Paramedic team members rallied as active followers, which participants described as strengthening team functioning through proactive support and activity.

*“Knowing that I have immediate access to experienced professionals boosts my confidence in handling the situation effectively. This reassurance helps [me] maintain composure and focus, which is crucial during emergencies.”*

Working alongside PRECARE teams has forged an ongoing impression and imbuing of confidence. Following the real-time mentoring and ensuing clinical debrief sessions, participants felt refreshed, valued and better informed about optimal OHCA care. Participants believed that they already had or would perform to a higher standard when attending subsequent prehospital cases.

*“The crews walked away from that job empowered by increased knowledge and confidence that I believe could have only been provided by an incredibly supportive medical team.”*

*“PRECARE has given me the confidence to trust in my skills and take a more proactive, evaluative approach, and I’ve noticed a similar shift in the workforce as well.“*

Working alongside PRECARE teams prompted participants to reflect more closely on their clinical responsibilities. Respondents felt encouraged to perform their clinical responsibilities to even higher standards. Furthermore, they reported improved efforts to consciously monitor the effectiveness of administered interventions to achieve highest level care. This encouragement was not limited to technical skills either, with many participants reporting calculated efforts to improve their non-technical skills (NTS) and management of relevant human factors, such as leadership and communication.

*“Working with the PRECARE team has strengthened my motivation to do my role well and get the ‘basics’ done well.”*

*“By seeing in real time the impact of the [PRECARE] team’s interventions, it has reignited a fire in us all to optimise our care in the future.”*

Participants felt inspired to undertake self-directed learning to improve their understanding of OHCA and the rationale supporting current prehospital and in-hospital treatments. This motivation was widespread with many participants reporting coordinated learning amongst friend and colleague groups.

*“The PRECARE team’s commitment to continuous improvement and learning has inspired me to adopt a similar mindset. This includes regularly seeking feedback, reflecting on experiences, and striving for personal and professional growth.”*

*“It has led my friends and I to go back into the textbooks and to research on paper and online how [we] can better improve [our] cardiac arrest care in the future.”*

Together, these accounts suggest that exposure to experienced clinicians during high-acuity cases reinforced professional confidence and encouraged participants to engage more actively with both technical and non-technical aspects of OHCA management.

### Theme three: understanding and development

This theme captures how interactions with PRECARE clinicians deepened participants’ conceptual understanding of cardiac arrest physiology and resuscitation science.

The real-time mentoring and debrief sessions provided by PRECARE clinicians were described as reinforcing and extending participants’ existing knowledge base. Participants reported a refreshed understanding of the pathophysiology of OHCA and the supporting evidence for relevant treatments. PRECARE teams also taught participants about possible atypical physiological presentations amongst OHCA populations, and the differences between cardiac arrest and physiological low-flow states.

*“The PRECARE team has significantly enhanced my understanding of the physiology of cardiac arrest and the importance of tailored, evidence-based interventions. Their involvement has deepened my comprehension of the underlying mechanisms during resuscitation, and I now have a better grasp of how specific treatments impact outcomes.”*

*“PRECARE provided us with information post-arrest regarding variations to the anatomy of the patient that were reducing the effectiveness of our compressions.”*

Similarly, many participants reported a renewed and deeper appreciation for the physiological rationale underpinning BLS interventions. This extended to HPCPR, mCPR application and ALS practises.

*“It’s made me far more vigilant about effective CPR, particularly [when recruiting] non-medical bystanders.”*

*“The PRECARE team engagement has increased my understanding and practical application of micro skills, such as improving the positioning of mCPR devices, to improve circulation.”*

Respondents also described a refreshed appreciation for physiological monitoring during OHCA resuscitation, not only to observe trends in vital signs but also to evaluate the effectiveness of clinical interventions.

*“I’ve also taken away an increased awareness of the need to cheque the efficacy of my treatments when managing cardiac arrests. I know what high performance CPR looks like, and I know how to do it, but I feel I now have a better understanding of how to cheque if the CPR is producing the desired result.”*

*“I’ve learned to better incorporate advanced monitoring and diagnostic tools such as EtCO_2_ which helps in quickly assessing a patient’s condition and tailoring the response accordingly.”*

Participants recognised themselves as being adequately trained in high-quality BLS, though after working alongside PRECARE teams they identified areas of opportunity to further their understanding of OHCA pathophysiology and resuscitation. Provided examples included consideration of the pathophysiology behind reversible causes of arrest and physiological low-flow states and practising non-technical skills (NTS) such as leadership, decision making and communication.

*Working alongside PRECARE has highlighted several personal and team-wide needs relating to cardiac arrest management… PRECARE has helped me see the importance of simple but often overlooked practises—like checking that interventions are truly effective and reassessing patients after each step*…*”*

Collectively, these reflections indicate that interactions with PRECARE clinicians reinforced existing knowledge while prompting participants to revisit foundational physiological concepts underpinning resuscitation practise.

### Theme four: cause-directed care

This theme reflects how participants translated this knowledge into more individualised and cause-directed approaches to OHCA management.

Participants expressed concerns that OHCA management, though well-rehearsed within the service, had previously felt protocol-driven, *“robotic”* and generalised. Many described OHCA treatment goals to be condensed to *“going through the motions*” until either ROSC is achieved or the patient is declared deceased. However, following discussions with PRECARE team members, participants reported a deeper appreciation for the need for an individualised, nuanced and holistic approach to these cases.

*“My experience with PRECARE influenced how I make patient-centred decisions when managing cardiac arrests. I better appreciate that patients can present with unique complications and may require individualised strategies to address their specific issues or pathology.”*

*“The PRECARE team has profoundly influenced my appreciation of the nuanced, patient-centred approach to cardiac arrest management.”*

After working with PRECARE teams, participants described a renewed emphasis on recognising and addressing potentially reversible causes of OHCA. This was reported as something which had been underemphasised during previous paramedic training, having been overshadowed by HPCPR drills, such as COACHED (17). Participants expressed a desire to integrate this cause-directed approach into their OHCA management practises, believing that it will result in better patient outcomes.

*“Interactions with the PRECARE team have encouraged me to think earlier and more deeply about reversible causes of cardiac arrest and how we can adjust our treatment to adequately address the identified cause of arrest.”*

*“PRECARE has influenced my ability to prioritise on cardiac arrest jobs through really focusing on identifying and treating reversible causes as early as possible rather than settling into a repetitive process on every arrest.”*

Paramedic leaders expressed a desire to be more flexible and adaptive in their crew resource management practises when attending OHCA cases to better facilitate this cause-directed care. This evolution reflected a growing capacity to apply knowledge and skills fluidly in complex and changing situations. To achieve this, these participants reported modifying their existing NTS, such as communication, to better create and delegate shared tasks in effort to provide more effective team-based cause-directed care.

*“From a team leader and human factors point of view, setting the course trajectory early on and sharing that with the crew I feel has been beneficial. For example, identifying that an arrest has a likely reversible cause that can be treated at a hospital and we should orientate ourselves to make that happen early on*…*”*

*“As a result of these debriefs, as a station we have looked at the guidelines and outlined the importance of targeting reversible causes and not being robotic in cardiac arrest management.”*

Collectively, these perspectives suggest a shift from protocol-driven resuscitation towards a more individualised approach.

### Theme five: collaborative care

This theme explores how interdisciplinary collaboration influenced team dynamics and non-technical skill development.

Working with senior interdisciplinary teams provided opportunities for paramedics to observe the NTS and human factors management required to establish a harmonious working environment and maintain effective teamwork. Participants recognised emotional intelligence, person-centred leadership, active followership, and communication skills as being particularly important. Participants expressed a desire to improve their own NTS to better coordinate teams and achieve team goals themselves.

*“I was impressed by how the [PRECARE clinician] was able to create an environment where all clinicians felt comfortable to speak up, perform their own assessments and interventions, and discuss ideas…”*

*“By working closely with the entire team, I’ve learned how effective it is to engage everyone’s input and perspectives in order to optimise patient care.”*

*“I’ve taken a lot of insight on crew resource management from watching the team work efficiently and learning to emulate a similar workflow.”*

Participants’ confidence in applying NTS into their future practise extended beyond interactions with other paramedic teams. Through observing and engaging with PRECARE clinicians, participants developed a deeper appreciation for effective interprofessional collaboration in the delivery of optimal care in OHCA scenarios.

*“Working with PRECARE has expanded my professional network, allowing me to collaborate with a diverse group of healthcare professionals. This has fostered a greater sense of community and shared purpose in improving emergency medical care.”*

*“Collaborating with other healthcare professionals, such as doctors and emergency room staff, can enhance our understanding of the entire cardiac arrest management process and improve overall team dynamics.”*

Some participants expressed concerns regarding the integration of PRECARE clinicians into paramedic teams, particularly during the early phases of the PRECARE trial. These accounts suggested that the introduction of a new interdisciplinary team initially created uncertainty around roles, leadership dynamics, and the decision-making authority. Participants described situations in which the responsibilities of paramedic team leaders and PRECARE clinicians were not yet clearly established, contributing to perceptions of an “us and them” dynamic.

*“I have done more than one PRECARE job and the first one I attended was in the infancy of PRECARE and I found the first job was very much us and them, and there was not much support mostly because I believe we were both not really sure of what each of our roles were. But in my second PRECARE job the trial was in it’s second year and I felt we both supported each other a lot better.”*

*“I stated early on that the leadership role can get a little blurred at first but I have found that has improved and I think that’s a change in approach from both perspectives.”*

These reflections highlight the importance of clearly defined leadership structures and deliberate NTS strategies when integrating new clinical roles into established team environments. Participants’ observations that these dynamics improved over time suggest that interdisciplinary collaboration evolves as teams develop shared expectations, communication patterns, and a mutual understanding of roles and responsibilities.

Some participants also raised concerns about aspects of PRECARE operational practise, particularly prolonged scene times associated with advanced procedures. These comments reflect tensions between traditional Anglo-American-styled EMS priorities, such as rapid transport,^17^ and the PRECARE model’s emphasis on advanced diagnostics and interventions delivered in the prehospital environment.

*“… Having done a few jobs with the PRECARE team, it has shown me time after time that the only contribution the PRECARE team has to the cardiac arrest is a significant prolongation in scene time without actually achieving anything meaningful…”*

*“I sometimes scratch my head in regards to why we need to spend an extra half an hour on scene with a cardiac arrest patient that has a ROSC, just so the PRECARE team can put an arterial line in.”*

These perspectives illustrate how the introduction of advanced critical care capabilities into the prehospital setting can challenge existing mental models of OHCA management. They highlight the importance of communication, shared clinical reasoning, and ongoing education to ensure that paramedic teams understand the rationale behind new diagnostic and therapeutic approaches.

These findings highlight how interdisciplinary collaboration can influence team dynamics and expose paramedics to alternative approaches to leadership, communication, and shared decision-making.

In summation, these themes suggest that working alongside PRECARE teams was perceived to influence paramedic practise through mentorship, strengthened confidence, deeper understanding of OHCA care, more cause-directed decision-making, and exposure to interdisciplinary team dynamics. At the same time, some accounts highlighted tensions related to role clarity and operational priorities, underscoring the complexity of integrating specialist teams into established EMS systems.

## Discussion

This study explored how specialist interdisciplinary cardiac arrest teams can influence paramedic practise and professional development. The findings largely suggest that real-time interdisciplinary collaboration, supported by mentorship and structured reflection, offers a strong foundation for paramedic learning and growth.

Experiential learning, characterised by the acquisition of knowledge and skills through direct engagement in clinical situations,^18^ was recognised as foundational to professional development. Working alongside PRECARE clinicians, paramedics observed expert practise and participated in complex decision-making within real-world, high-stakes environments. PRECARE team members modelled expert clinical reasoning and provided real-time mentorship, supporting the translation of theoretical knowledge into practise and refinement of both technical and non-technical skills. Paramedics reported increased confidence, enhanced capability, and greater reactivity and adaptability. This mentorship enabled learning that extended beyond what is typically achieved through simulation or classroom instruction alone. These findings reflect existing literature showing that experiential learning, especially when paired with expert guidance, strengthens reasoning, promotes skill development, and improves readiness for holistic high-acuity care.^18^, ^19^, ^20^

This learning was made possible by the presence of psychological safety. Defined as the shared belief that team members can express ideas, seek and offer honest feedback, and take interpersonal risks without fear of embarrassment or retribution,^21^ psychological safety is associated with improved team performance, patient outcomes, and learning.^22^, ^23^, ^24^ Participants described how the approachable, respectful conduct of PRECARE clinicians fostered a culture of openness and trust. This environment encouraged paramedics to ask questions, contribute to discussions, and reflect critically without concern for negative judgement. These conditions made it easier to share ideas, have meaningful discussions, and apply feedback to improve practise. In this way, psychological safety served as a foundational enabler of experiential learning.

Participants viewed reflective debrief sessions as structured opportunities for collaborative sense making, reviewing decisions, exploring alternatives, and consolidating knowledge. When situated in psychologically safe spaces, debriefs supported the integration of experience with clinical reasoning, strengthening confidence and critical thinking.^25^, ^26^, ^27^, ^28^ Being interprofessional in nature was particularly valuable because it exposed paramedics to diverse clinical reasoning approaches. Rather than focusing solely on outcomes, these sessions explored decision rationale, examined team dynamics and clarified roles. These findings align with literature highlighting the value of debriefing in reinforcing shared mental models, improving collaboration, and promoting reflective practise.^25^, ^27^, ^29^ In paramedicine and other high-acuity settings, debriefs are associated with increased clinical preparedness, more effective decision-making under pressure, and greater confidence in managing time-critical scenarios.^25^, ^27^, ^28^, ^30^ By fostering reflective practise and interdisciplinary insight, they also contribute to the development of professional identity.^31^, ^32^

Collaboration with senior clinicians broadened participants’ understanding of patient trajectories beyond their immediate prehospital encounter, illustrating how paramedic decisions can shape longer-term outcomes. Through interprofessional mentorship, paramedics gained clinical knowledge, systems thinking, clearer role delineation, and improved collaborative decision-making. These findings reflect literature on interprofessional education which highlights the value of diverse perspectives in enhancing communication, teamwork, and confidence.^28^, ^33^, ^34^ Within paramedicine, such collaboration supports integrated care models, strengthens care transitions, and prepares clinicians to work across organisational and disciplinary boundaries.^29^, ^35^ However, participants’ experiences were not uniformly positive. Whilst most participants described PRECARE involvement positively, some accounts highlighted tensions related to role clarity, leadership dynamics, and operational priorities. These observations underscore the importance of deliberate NTS and human factor strategies when integrating new interdisciplinary roles into established EMS teams. These findings may also reflect contrasts between participants’ routine experiences of OHCA management and the practises demonstrated by a specialist response team, suggesting that exposure to high-performing interdisciplinary teams can highlight opportunities for practise development within existing systems.

Altogether, these findings have important implications for paramedic education and workforce development. Specialist interdisciplinary teams such as PRECARE may provide a powerful model of real-world learning that supports skill acquisition, collaborative practise, and systems-level thinking. Formalising such models within paramedic education could significantly enhance professional growth. More broadly, the PRECARE experience underscores the value of embedding interprofessional educators within ambulance services. Real-time collaboration with clinicians from diverse disciplines promotes systems literacy, team competence, and reflective practise. Investing in these roles is a strategic step toward building a more collaborative and future-ready paramedic workforce.

More broadly, these findings align with the 2025 International Liaison Committee on Resuscitation (ILCOR) Consensus in Science with Treatment Recommendations (CoSTR) which advises the dispatch of critical care teams to non-traumatic OHCA within EMS systems with sufficient resource infrastructure. The CoSTR also emphasises the importance of structured post-event debriefing for teams who attend adult, paediatric and neonatal OHCA, and recommends the integration of a CPR coach as a member of prehospital resuscitation teams.^36^ These recommendations are already embedded within the PRECARE model and are supported by the results of this study.

These findings should be interpreted with consideration of several limitations. As an interpretive qualitative study, findings are contextually bound and not intended to be statistically generalisable. While transferability to similar interdisciplinary settings is possible, caution is needed when applying results to different systems or team structures. The use of an online open-ended survey potentially limited the depth of responses compared to interviews or focus groups. However, the authors considered theoretical sufficiency to have been achieved based on the scope of the research question, the richness of the dataset, and the depth and coherence of the developed themes. The survey response rate was 17.8%; however, response rates themselves are not indicative of quality in qualitative research. Unlike quantitative studies, which seek statistical representativeness, qualitative research prioritises depth and richness of insight, and it is the quality of the data, rather than the quantity of responses, that underpins qualitative analytical rigour.^37^, ^38^

## Conclusions

PRECARE teams offered paramedics more than real-time clinical support. Their presence fostered psychological safety, collaborative learning, and meaningful post-event reflection. Through guided experiential learning, paramedics developed greater confidence, refined clinical reasoning, and a shift toward more nuanced, cause-directed care. Furthermore, interdisciplinary collaboration broadened paramedics’ understanding of patient trajectories and system-level factors, reinforcing integrated care approaches. Together, these elements were perceived to enhance individual paramedic capability, reinforce team effectiveness, and may have implications for the quality of out-of-hospital resuscitation care. At the same time, some participants described challenges related to role clarity, leadership dynamics, and alignment of operational priorities, reinforcing the need for clearly defined roles and a shared understanding of team function when integrating specialist teams into established EMS systems.

## CRediT authorship contribution statement

**Jacob Tant:** Writing – review & editing, Writing – original draft, Project administration, Methodology, Investigation, Formal analysis, Data curation, Conceptualization. **Jackie Buckthought:** Writing – review & editing, Data curation, Conceptualization. **Ashleigh Scianski:** Writing – review & editing, Writing – original draft, Formal analysis. **Izabelle Xu:** Writing – review & editing, Data curation. **Brian Burns:** Writing – review & editing, Supervision. **Natalie Kruit:** Writing – review & editing, Supervision, Conceptualization.

## Funding

This study did not receive any funding.

## Declaration of competing interest

The authors declare that they have no known competing financial interests or personal relationships that could have appeared to influence the work reported in this paper.

## References

[1] Richardson S.A.C., Anderson D., Burrell A.J.C. (2023). Pre-hospital ECPR in an Australian metropolitan setting: a single-arm feasibility assessment—the CPR, pre-hospital ECPR and early reperfusion (CHEER3) study. Scand J Trauma Resusc Emerg Med.

[2] Boulton A.J., Edwards R., Gadie A. (2024). Prehospital critical care beyond advanced life support for out-of-hospital cardiac arrest: a systematic review. Resusc Plus.

[3] Kruit N., Burns B., Shearer N. (2025). Pre-hospital ECPR for refractory cardiac arrest – the PRECARE pilot feasibility study. Resuscitation.

[4] Leroux L., Dennis-Benford N.B., Bergeron A. (2025). Impact of prehospital extracorporeal cardiopulmonary resuscitation for out-of-hospital cardiac arrest on survival with good neurological function: a systematic review and meta-analysis. Resusc Plus.

[5] New South Wales Ambulance (2024). https://www.ambulance.nsw.gov.au/__data/assets/pdf_file/0006/982878/DE886-Year-in-Review-2023-2024.pdf.

[6] New South Wales Ambulance (2023). https://www.ambulance.nsw.gov.au/__data/assets/pdf_file/0006/971853/NSW-Ambulance-OHCAR-2022-Report.pdf.

[7] Braun V., Clarke V. (2013).

[8] Braun V., Clarke V. (2017). Thematic analysis. J Posit Psychol.

[9] Braun V., Clarke V. (2020). Can I use TA? Should I use TA? Should I not use TA? Comparing reflexive thematic analysis and other pattern-based qualitative analytic approaches. Couns Psychother Res.

[10] O'Brien B.C., Harris I.B., Beckman T.J., Reed D.A., Cook D.A. (2014). Standards for reporting qualitative research: a synthesis of recommendations. Acad Med.

[11] Simpson P. (2025). Doing better with survey-based research in paramedicine. Paramedicine.

[12] Thomas S.L., Pitt H., McCarthy S., Arnot G., Hennessy M. (2024). Methodological and practical guidance for designing and conducting online qualitative surveys in public health. Health Promot Int.

[13] Terry G., Hayfield N., Clarke V., Braun V., Willig C., Rogers W.S. (2017). The SAGE handbook of qualitative research in psychology.

[14] Byrne D. (2021). A worked example of Braun and Clarke’s approach to reflexive analysis. Qual Quant.

[15] Guest G., Bunce A., Johnson L. (2006). How many interviews are enough?. Field Methods.

[16] National Health and Medical Research Council, Australian Research Council, Universities Australia (2018). https://www.nhmrc.gov.au/about-us/publications/national-statement-ethical-conduct-human-research-2007-updated-2018.

[17] Makrides T., Ross L., Gosling C., Acker J., O’Meara P. (2022). From stretcher bearer to practitioner: a brief narrative review of the history of the Anglo-American paramedic system. Austr Emerg Care.

[18] Ross L.J., Jennings P.A., Gosling C.M. (2018). Experiential education enhancing paramedic perspective and interpersonal communication with older patients: a controlled study. BMC Med Educ.

[19] Melby V. (2000). Experiential learning in pre-hospital emergency care. Nurse Educ Today.

[20] Mangan J., Rae J., Anderson J. (2022). Undergraduate paramedic students and interpersonal communication development: a scoping review. Adv Health Sci Educ.

[21] Edmondson A. (1999). Psychological safety and learning behavior in work teams. Adm Sci Q.

[22] Lockhart A., Walker T., Bowles K.-A. (2024). A qualitative study on the relationship between leadership and clinical skills in paramedicine and the promotion of patient safety. Paramedicine.

[23] Purdy E., Borchert L., El-Bitar A. (2023). Psychological safety and emergency department team performance: a mixed-methods study. Emerg Med Australas.

[24] Grailey K.E., Murray E., Reader T. (2021). The presence and potential impact of psychological safety in the healthcare setting: an evidence synthesis. BMC Health Serv Res.

[25] Fanning R.M., Gaba D.M. (2007). The role of debriefing in simulation-based learning. Simul Healthc.

[26] Tannenbaum S.I., Cerasoli C.P. (2013). Do team and individual debriefs enhance performance? A meta-analysis. Hum Fact.

[27] Tierney S. (2018). The utilisation of a structured debriefing framework within the pre-hospital environment: a service evaluation. Br Paramed J.

[28] Avery P., Thompson C., Cowburn P. (2023). Training the trainers: improving the quality of education delivered to paramedics through a simulation-debrief model. Br Paramed J.

[29] Mulholland P., Barnett T., Woodroffe J. (2019). A grounded theory of interprofessional learning and paramedic care. J Interprof Care.

[30] Paxino J., Szabo R.A., Marshall S. (2024). What and when to debrief: a scoping review examining interprofessional clinical debriefing. BMJ Qual Saf.

[31] Thompson J., Couzner L., Houston D. (2020). Assessment partnerships from the start: building reflective practice as a beginning paramedic student competency. Austr J Paramed.

[32] He Q., Dizon J., Tipoe G.L. (2025). Exploring pathways to develop interprofessional identity: a moderated mediation study. Adv Health Sci Educ.

[33] Mulholland P., Barnett T., Spencer J. (2014). Interprofessional learning and rural paramedic care. Rural Remote Health.

[34] Furseth P.A., Taylor B., Kim S.C. (2016). Impact of interprofessional education among nursing and paramedic students. Nurse Educ.

[35] Andrews G., Davis K., Govind N., MacLellan A., Kerrison-Watkin G., Patel H. (2025). Learning together, responding together: interprofessional learning enhancing emergency services collaboration. Health Educ Pract.

[36] Berg K.M., Bray J.E., Djärv T. (2025). Executive summary: 2025 International Liaison Committee on Resuscitation Consensus on Science With Treatment Recommendations. Circulation.

[37] LaDonna K.A., Artino A.R., Balmer D.F. (2021). Beyond the guise of saturation: rigor and qualitative interview data. J Grad Med Educ.

[38] Sandelowski M. (1995). Sample size in qualitative research. Res Nurs Health.

