# Supplementary material for: The impact of interdisciplinary cardiac arrest teams on paramedic practise: an interpretive qualitative study using reflexive thematic analysis
Source: Resusc Plus. 2026 Apr 7;29:101317. doi: 10.1016/j.resplu.2026.101317 (PMC13123611; doi:10.1016/j.resplu.2026.101317)
Supplement: Supplementary Table 3 [file mmc1.docx]

**Appendix**

**Table 3:** Individual participant demographic details.

| **Participant Number (P#)** | **Paramedic Qualifications** | **Years of Paramedic Experience** | **Highest Level of Education** | **Estimated OHCA Cases Attended per Year** |
| --- | --- | --- | --- | --- |
| P1 | ICP | 0–5 | Master's degree | 12 |
| P2 | Paramedic | 0–5 | Postgraduate certificate or degree | 5 |
| P3 | ICP | 6–10 | Postgraduate certificate or degree | 30 |
| P4 | Paramedic | 0–5 | Bachelor's degree | 30 |
| P5 | ICP, Extended care paramedic (ECP) | 11–15 | Postgraduate certificate or degree | 6 |
| P6 | ICP | 6–10 | Master's degree | 15 |
| P7 | ICP, ECP | 21–25 | Master's degree | 6 |
| P8 | Paramedic | 6–10 | Bachelor's degree | 10 |
| P9 | ICP | 11–15 | Master's degree | 20 |
| P10 | Paramedic | 0–5 | Bachelor's degree | 3 |
| P11 | ICP | 11–15 | Bachelor's degree | 25 |
| P12 | ICP | 6–10 | Bachelor's degree | 25 |
| P13 | ICP | 16–20 | Postgraduate certificate or degree | 10 |
| P14 | ICP | 0–5 | Bachelor's degree | 7 |
| P15 | ICP | 6–10 | Postgraduate certificate or degree | 25 |
| P16 | Paramedic | 0–5 | Bachelor's degree | 6 |
| P17 | ICP | 6–10 | Bachelor's degree | 20 |
| P18 | ICP | 6–10 | Bachelor's degree | 36 |
| P19 | ICP | 26–30 | Bachelor's degree | 11 |
| P20 | ICP | 26–30 | Bachelor's degree | 10 |
| P21 | Paramedic | 16–20 | Bachelor's degree | 10 |
| P22 | ICP, ECP, Special Operations / Rescue | 26–30 | Bachelor's degree | 6 |
| P23 | Paramedic | 0–5 | Postgraduate certificate or degree | 6 |
| P24 | ICP | 36–40 | Associate degree | 20 |
| P25 | Paramedic | 11–15 | Associate degree | 2 |
| P26 | ICP, ECP | 16–20 | Postgraduate certificate or degree | 10 |
| P27 | Paramedic | 0–5 | Bachelor's degree | 10 |
| P28 | ICP | 21–25 | Associate degree | 20 |
| P29 | ICP | 6–10 | Bachelor's degree | 55 |
| P30 | Paramedic | 0–5 | Bachelor's degree | 8 |
| P31 | Paramedic | 0–5 | Bachelor's degree | 5 |
| P32 | Paramedic | 16–20 | Associate degree | 10 |
| P33 | ICP | 26–30 | Postgraduate certificate or degree | 7 |
| P34 | Paramedic | 0–5 | Bachelor's degree | 5 |
| P35 | Paramedic | 0–5 | Bachelor's degree | 5 |
| P36 | Trainee paramedic | 0–5 | Bachelor's degree | 1 |
| P37 | ICP | 16–20 | Postgraduate certificate or degree | 6 |
| P38 | Paramedic | 0–5 | Bachelor's degree | 6 |
| P39 | ICP | 11–15 | Associate degree | 11 |
| P40 | Paramedic | 6–10 | Bachelor's degree | 14 |
| P41 | ICP | 21–25 | Bachelor's degree | 50 |
| P42 | ICP | 6–10 | Postgraduate certificate or degree | 20 |
| P43 | ICP | 6–10 | Bachelor's degree | 20 |
| P44 | ICP | 21–25 | Postgraduate certificate or degree | 10 |
| P45 | ICP | 6–10 | Bachelor's degree | 15 |
| P46 | ICP | 16–20 | Associate degree | 25 |
| P47 | ICP | 26–30 | Postgraduate certificate or degree | 28 |
| P48 | ICP | 6–10 | Bachelor's degree | 35 |
| P49 | ICP, ECP | 6–10 | Postgraduate certificate or degree | 52 |
| P50 | ICP, ECP | 11–15 | Bachelor's degree | 15 |
| P51 | ICP | 6–10 | Master's degree | 12 |
| P52 | Paramedic | 0–5 | Bachelor's degree | 7 |
| P53 | ICP | 16–20 | Bachelor's degree | 50 |
| P54 | ICP | 6–10 | Postgraduate certificate or degree | 15 |
| P55 | ICP | 11–15 | Postgraduate certificate or degree | 30 |
| P56 | ICP, ECP | 6–10 | Postgraduate certificate or degree | 20 |
| P57 | ICP | 16–20 | Bachelor's degree | 20 |
| P58 | ICP | 11–15 | Postgraduate certificate or degree | 40 |
| P59 | ICP, ECP | 11–15 | Bachelor's degree | 12 |
| P60 | ICP | 6–10 | High school | 8 |
| P61 | Paramedic | 16–20 | Associate degree | 30 |
| P62 | ICP | 11–15 | Bachelor's degree | 25 |
| P63 | ICP | 11–15 | Bachelor's degree | 20 |
| P64 | Paramedic | 0–5 | Bachelor's degree | 8 |
| P65 | Paramedic | 0–5 | Bachelor's degree | 10 |
| P66 | Paramedic | 0–5 | Bachelor's degree | 15 |
| P67 | ICP | 26–30 | Associate degree | 16 |
